# Epidemiology and antibiotic resistance of staphylococci on commercial pig farms in Cape Town, South Africa

**DOI:** 10.1038/s41598-024-70183-2

**Published:** 2024-08-26

**Authors:** Remous Ocloo, Mae Newton-Foot, Lucious Chabuka, Wilma Ziebuhr, Andrew Christopher Whitelaw

**Affiliations:** 1 Department of Pathology, Division of Medical Microbiology, Stellenbosch University, Stellenbosch, South Africa; 2 National Health Laboratory Service (NHLS), Tygerberg Hospital, (TBH), Cape Town, South Africa; 3 Centre for Epidemic Control and Innovation (CERI), School of Data Science and Computational Thinking, Stellenbosch University, Stellenbosch, South Africa; 4 Public Health Institute of Malawi, Ministry of Health, Lilongwe, Malawi; 5 Institute of Molecular Infection Biology, Würzburg University, Würzburg, Germany; 6 TASK, Cape Town, South Africa

**Keywords:** Microbiology, Molecular biology

## Abstract

Staphylococci are responsible for a wide range of infections in animals. The most common species infecting animals include *Staphylococcus aureus, Staphylococcus epidermidis* and *Staphylococcus intermedius.* Recent increases in antibiotic use and antibiotic resistance in animals highlight the need to understand the potential role of commercial livestock as a reservoir of staphylococci and antibiotic resistance genes. Nasal swabs were collected from 143 apparently healthy pigs and 21 pig farm workers, and 45 environmental swabs of feed and water troughs, from two commercial pig farms in the Western Cape, South Africa. Staphylococci were isolated, identified using mass-spectrometry, and antimicrobial susceptibility testing and Illumina whole genome sequencing were performed. One hundred and eighty-five (185) S*taphylococcus* spp. isolates were obtained, with *Mammalicoccus sciuri* (*n* = 57; 31%) being the most common, followed by *S. hyicus* (*n* = 40; 22%) and *S. aureus* (*n* = 29; 16%). *S. epidermidis* was predominantly identified in the farm workers (n = 18; 86%). Tetracycline resistance was observed across all species, with rates ranging from 67 to 100%. Majority *of M. sciuri* isolates (*n* = 40; 70%) were methicillin resistant, with 78% (*n* = 31) harbouring *mecA*. *M. sciuri* isolates had genes/elements which were associated with SCC*mec*_type_III (3A) and SCC*mec*_type_VIII(4A) and were mostly observed in ST61 strains. ST239 strains were associated with SCC*mec*_type_III(3A). High rates of tetracycline resistance were identified among staphylococci in the pig farms in Western Cape, South Africa. This highlights the need for policy makers to regulate the use of this antibiotic in pig farming.

## Introduction

Staphylococci are commensals and opportunistic pathogens in animals, responsible for a wide range of infections such as mastitis in cows and exudative epidermitis in pigs^1^. The common species colonizing and infecting animals include *Staphylococcus aureus*, *Staphylococcus epidermidis, Staphylococcus intermedius, Staphylococcus xylosus, Staphylococcus chromogenes, Staphylococcus hyicus, Staphylococcus hominis,* and *Staphylococcus haemolyticus*^2,3^.

Much of the research on staphylococci in animals has focused on *S. aureus* due to its importance as a human pathogen. *S. aureus* mostly causes mastitis in cattle and pyoderma in dogs and cats^4,5^, while pigs are mostly asymptomatic carriers^6^. A review on *S. aureus* in animals and food in Africa suggests that the prevalence of methicillin susceptible *S. aureus* (MSSA) in animals ranges from 3 to 58% depending on the type of animal and the source of sample^2^. The colonization of animals by methicillin resistant *S. aureus* (MRSA) in Africa was found to be very low, ranging from 0 to 3%^2^. Livestock-Associated Methicillin-Resistant *Staphylococcus aureus* (LA-MRSA) is an emerging pathogen globally. The average carriage rate of LA-MRSA in European countries is 14% in pig breeding farms and 26.9% in pig production farms^7^. A typical LA-MRSA strain is sequence type (ST) 398, which has been reported to cause infections in humans, particularly in individuals with frequent contact with animals^8^. Recently, ST9 has been reported to be an emerging LA-MRSA strain in Asia^9^ There have also been reports of human to animal transmission of *S. aureus* across the globe^7,10,11^.

Little is known about the prevalence and epidemiology of Staphylococcus other than *S. aureus* (SOSA), especially in Africa^3^. A recent systematic review on SOSA in domestic animals and livestock in Africa showed that most research on SOSA in animals has focused on cattle (32/75 studies)^3^. However, pigs are one of the most common livestock globally and in South Africa^12,13^. Staphylococci other than *S. aureus* such as *S. haemolyticus* have been reported in pigs from Nigeria^14^ and South Africa^15^. Only a few cases of SOSA transmission between humans and animals have been reported globally^16,17^. However, there are few studies describing zoonotic and reverse zoonotic transmission on pig farms in Sub-Saharan Africa^9^.

In South Africa, the pig population was estimated at 1.357 million for the year 2020, and the Western Cape province accounts for 11% of South Africa’s pig production^13^. Considering the high usage of antibiotics, especially tetracycline, in pig production in South Africa^18^, the lack of data on antibiotic resistance in pig-associated SOSA globally and in South Africa is of concern. Similarly, there is little information on the population structure and antibiotic resistance mechanisms in LA-SOSA in South Africa. Therefore, there is a need to understand the potential role of pig farms as a reservoir of staphylococci and antibiotic resistance genes^19^.

This study describes the species distribution, population structure and antibiotic resistance mechanisms amongst staphylococci from pigs, their caregivers, and the pig farm environment in the Western Cape, South Africa.

## Methods

### Study setting

The study was conducted at two commercial pig farms (Farm A and Farm B) in the Western Cape Province of South Africa; both farms have herd sizes of between 500 and 1000 pigs. Farm A is a high health pig farm while Farm B is a standard health farm*.* High health farms have strict biosafety measures in place to limit the introduction and spread of infectious diseases. These include strict access control and regular disease monitoring and surveillance. Standard health farms have less stringent biosafety measures in place.

### Study Population and sample collection

The study population consisted of the pigs on the farms as well as farm workers. Samples were collected from all farm workers who have direct or indirect contact with the pigs and gave consent. Bio-Cult Amies transport swabs with charcoal (Liofilchem, Italy) were used to obtain nasal swabs from farm workers (self-collected from the anterior nares), as well as for sampling the nostrils of the pigs. At least 10 pigs were sampled per pen/holding on each farm. Environmental samples were collected from feed, water troughs and sewage at each farm. Demographic data such as age, sex, antibiotic use and living conditions were collected from the farm workers. The farm managers provided information on antibiotic use and hygiene practices on the farms (Supplementary S1).

### Isolation and identification of staphylococci

The swabs were stored at 4 °C for ≤ 4 days before analysis. The swabs were inoculated in 2 ml Lysogeny Broth and incubated aerobically for 6 h at 37 °C to promote the growth of staphylococci. The broth was then sub-cultured onto 5% sheep blood agar plates supplemented with 10 μg/ml each of nalidixic acid and colistin (Thermofisher, USA) to suppress the growth of gram-negative bacteria. Isolates were selected based on a mostly creamy colony morphology consistent with staphylococci. Multiple isolates were collected from each individual, an approach that enabled us to describe the distribution of different staphylococcal species colonizing the pigs and the farm workers.

Presumptive staphylococci were further confirmed based on a negative bile esculin agar test and a positive catalase test (both from NHLS Media Laboratory, Greenpoint, South Africa), and identified using matrix-assisted laser desorption/ionization time-of-flight mass spectrometry (MALDI-TOF/MS; VITEK® MS, BioMérieux, France).

### Antibiotic susceptibility testing

Antibiotic susceptibility testing (AST) was performed on *S. aureus* isolates and the most common SOSA species using Kirby Bauer disc diffusion. The following antibiotic discs (MAST Group, UK) were used: cefoxitin (30 µg), clindamycin (2 µg), erythromycin (15 µg), rifampicin (5 µg), linezolid (30 µg), trimethoprim/sulfamethoxazole (1.25/23.75 µg), ceftaroline (30 µg), chloramphenicol (30 µg), fusidic acid (10 µg), levofloxacin (5 µg), and tetracycline (30 µg). Cefoxitin (30 µg) was used to determine oxacillin susceptibility. Vancomycin susceptibility testing was performed using vancomycin-supplemented (6 µg/ml) brain heart infusion media (NHLS Media Laboratory).

Susceptibility testing results were interpreted using the Clinical and Laboratory Standards Institute (CLSI) guidelines, 2020 for staphylococci; but where available, species specific breakpoints were used, such as for *S. aureus* and *S. epidermidis*^20^. The term non-susceptible used in this study refers to the CLSI guidelines for resistant and intermediately resistant staphylococci. Multidrug resistance (MDR) is defined as non-susceptibility to at least one agent from at least three different antibiotic classes^21^.

### DNA extraction and whole genome sequencing

Whole genome sequencing was performed at the Centre for Epidemic Response and Innovation, Stellenbosch University, using Illumina DNA Library Prep kits and the NextSeq 2000 (Illumina). Raw sequence reads were automatically generated, checked for quality, trimmed, and de novo assembled using FastQC v0.11.7, Trimmomatic v0.39 and SPAdes v3.15.5 respectively^22–24^. The assembled genomes were submitted to rMLST (https://pubmlst.org/species-id) for species identification^25^. Genomes showing matches to multiple staphylococcal species were further examined using EZBioCloud2 for average nucleotide identity (ANI), with identities assigned based on ≥ 95% ANI^26^.

Prokka v1.14.5 was used for genome annotation for common staphylococcal species^27,28^. PubMLST (https://pubmlst.org/organisms) and SCCmecFinder (https://cge.cbs.dtu.dk/services/SCCmecFinder/) were used to predict sequence types (ST) and SCC*mec* types, respectively. Resistance genes were identified using Resistance Gene Identifier (RGI) v6.00^29^.

### Statistical analysis

The minimum number of samples to be collected (sample size) was estimated to be 163 using a 95% confidence level, an expected methicillin resistance prevalence of 12%^19^ and desired precision of 5%^30^ in order to describe the prevalence of methicillin-resistant *Staphylococcus* spp. Differences in the prevalence of SOSA species and antibiotic resistance rates between the two farms were calculated using Fisher exact and Chi squared tests where appropriate. Microsoft Excel was used for the analysis and p-values of < 0.05 were considered significant.

### Ethical considerations

Ethical approval was obtained from the Research Ethics Committee at Stellenbosch University. Informed consent was obtained from the farm workers and managers. The study was approved by the South African Department of Agriculture, Land Reform and Rural Development.

The study was conducted in accordance with the Declaration of Helsinki (2013), as well as South African and ICH GCP Guidelines and the Ethical Guidelines of the Department of Health. The study also observed relevant regulations by the South African Department of Agricultural, Land Reform and Rural development.

## Results

On farm A, nasal swabs were collected from nine (9) farm workers and 73 pigs, and 22 environmental samples were taken; while on farm B, nasal swabs were taken from 12 farm workers and 69 pigs, and 23 environmental swabs were collected.

In total, 142 pigs were sampled, with ages ranging from 7 to 35 weeks*.* All pigs on farm A were finishers aged 25–30 weeks while in farm B, 45 (34.5%) pigs were finishers, 10 weaners aged 7–14 weeks, and nine piglets aged 3–5 weeks (Supplementary S2). Both farms have concrete flooring and engage in the following practices: use commercial food, use antibiotics to prevent infections, health check animals at least every three months, diagnose the animals using farm workers and a veterinarian, and impregnate pigs using artificial insemination. Twenty-one (21) farm workers were included of whom 71% (n = 15) were male. The time workers spent on the farms ranged from 6 to 12 h per day (Supplementary S3).

### Distribution of Staphylococci

Ninety six of the 142 pigs (68%) were colonised with any *Staphylococcus* species*. Mammaliicoccus sciuri* (previously *Staphylococcus sciuri*) was the commonest (n = 47; 33%), followed by *S. hyicus* (n = 37; 26%) and *S. aureus* (n = 15; 11%) (Table 1). Additionally, 20 of the 21 farm workers (95%) were colonised with any *Staphylococcus* spp., with *S. aureus* (n = 6; 29%) and *S. epidermidis* (n = 6; 29%) being the commonest. One or more staphylococcal species were isolated from 24 of the 45 environmental samples (53%): *M. sciuri* (n = 9; 20%), *S. aureus* (n = 8; 18%), *S. hyicus* (n = 7; 16%), *S. haemolyticus* (n = 3; 7%), *S. chromogenes* (n = 2; 4%), and *S. epidermidis* (n = 2; 4%).

**Table 1:** Distribution of staphylococci on the two farms.

	Farm A			Farm B		
	Pigs N = 73 (%)	Farm workers N = 9 (%)	Environmental swabs N = 22 (%)	Pigs N = 69 (%)	Farm workers N = 12 (%)	Environmental swabs N = 23(%)
Any staphylococcus	60 (82)	8(89)	16 (73)	39 (56)	12 (100)	11 (48)
*S. aureus*	11 (15)	2 (22)	5 (23)	4 (6)	4 (33)	3 (13)
Any SOSA	57 (78)	6 (67)	11 (50)	35 (51)	12 (100)	8 (35)
*Mammaliicoccus scuiri*	29 (39)	1 (11)	5 (23)	18 (26)	0	4 (17)
*Staphylococcus hyicus*	24 (32)	0	3 (14)	13 (19)	0	0
*Staphylococcus haemolyticus*	12 (16)	0	3 (14)	0	0	0
*Staphylococcus chromogenes*	9 (12)	0	0	11(16)	0	3 (13)
*Staphylococcus epidermidis*	1 (1)	6 (67)	2 (9)	0	12 (100)	0
Other SOSA	10 (14)	0	3 (14)	5 (7)	3 (25)	5 (22)

*Other SOSA represents Staphylococcus xylosus, Staphylococcus mulans, Staphylococcus. warneri, Staphylococcus saprophyticus, Staphylococcus gallinarum, Staphylococcus cohnii, Staphylococcus lugdunensis.*

The prevalence of any staphylococcus was higher on farm A compared to farm B (*p* = *0.008*), however there were no statistically significant differences in the distribution of specific staphylococcal species between farms. This might be due to the small sample size. *S. epidermidis* was most common amongst farm workers (86%; n = 18) while *M. sciuri* (33%; n = 47) and *S. hyicus* (26%; n = 37) were common in pigs. *S. aureus*, and *M. sciuri* were common species isolated in the pigs, the farm workers and the environment.

### Antibiotic Susceptibility of SOSA

In total, 185 isolates were subjected to AST, and all (100%) were susceptible to chloramphenicol, linezolid, rifampicin, and vancomycin. All *S. chromogenes*, *S. hyicus*, and *S. haemolyticus* isolates were susceptible to oxacillin. All *M. sciuri* and *S. chromogenes* isolates were non-susceptible to clindamycin, and all *S. chromogenes* isolates were non-susceptible to tetracycline.

In isolates from the pigs, the rate of non-susceptibility to erythromycin ranged from 51 to 100%, to clindamycin from 59 to 100%, and to tetracycline from 93 to 100% between species (Table 2).

**Table 2:** Proportion of antibiotic non-susceptible staphylococci from pigs.

Species	Number of isolates	FOX n (%)	E n (%)	CD n (%)	TS n (%)	CPT n (%)	T n (%)	LEV n (%)	FA n (%)	MDR n (%)
*M. sciuri*	47	34 (72)	43 (91)	47 (100)	0	2 (4)	44 (94)	0	43 (91)	45 (96)
*S. hyicus*	37	0	19 (51)	22 (59)	3	0	36 (97)	1 (3)	0	19 (51)
*S. chromogenes*	21	0	12 (57)	21(100)	6 (29)	0	21(100)	0	0	2 (10)
*S. aureus*	15	1 (7)	12 (80)	15 (100)	0	0	14 (93)	8 (53)	0	12 (80)

*FOX* oxacillin, *TS* trimethoprim sulfamethoxazole, *CD* clindamycin, *E* erythromycin, *CPT* ceftaroline, *T* tetracycline, *LEV* levofloxacin, *FA* fusidic acid, *MDR* multidrug resistant.

A high proportion of the farm workers (n = 12; 57%) were also colonized by tetracycline non-susceptible *S. epidermidis.* Although only a small number of staphylococcal isolates were isolated from the environment, all isolates of all species were non-susceptible to tetracycline. Supplementary S4 shows the proportion of antibiotic resistance in SOSA isolated from farm workers and the environment.

### Whole genome sequence analysis of *Staphylococcus* spp.

The WGS of four *S. aureus* and one *S. hyicus* isolates had insufficient data and were excluded from further analysis, leaving 181 analysable isolates. Speciation of all isolates was confirmed by WGS analysis, except 3 of the 15 *S. haemolyticus* isolates which were identified as *Staphylococcus borealis* by WGS. The average nucleotide identity (ANI) of the three isolates was ≥ 95% when compared to *S. borealis* and ≤ 91% when compared to *S. haemolyticus.*

### Identification of antibiotic resistance genes

The penicillin resistance gene *blaZ* was common in both *S. aureus* (83%) and SOSA: *S. epidermidis* (90%), *S. chromogenes* (78%), *S. hyicus* (45%), and *S. haemolyticus* (20%) (Supplementary S5)*.* The *blaZ* gene was absent in all *M. sciuri*, however, the methicillin resistance gene *mecA* was more frequent in *M. sciuri* (54%) than other SOSA (20%). Of the 40-methicillin resistant *M. sciuri* isolates, 31 (78%) harboured *mecA*. Only one *S. epidermidis* was methicillin resistant and harboured the *mecA* gene. Only one methicillin susceptible *S. aureus* harboured *mecA.* A single MRSA isolate had neither *mecA* or *mecC.*

Although the majority of isolates across all species were non-susceptible to clindamycin, only seven *M. sciuri* and two *S. hyicus* harboured either *inuA* or *inuG.* The erythromycin resistance gene *ermC* was observed in *S. aureus* (23%) and across all SOSA species: *S. hyicus* (33%), *M. sciuiri* (33%), *S. chromogenes* (26%), *S. epidermidis* (14%), and *S. haemolyticus* (13%)*.* The tetracycline resistance gene *tet(K)* was also observed in *S. aureus* (46%) and across all SOSA species: *S. epidermidis* (67%), *S. haemolyticus* (67%)*, S. chromogenes* (39%), *S. hyicus* (33%) and *M. sciuiri* (12%). Other tetracycline resistance genes such as *tet(L)*, *tet(M)*, *tet(T)* and *tet(45)* were identified in the other isolates that were phenotypically tetracycline non-susceptible.

### Sequence typing and staphylococcal cassette chromosome *mec* typing

ST1 was the most common sequence type amongst *S. aureus* (n = 10; 61%) in farm A, while ST9 and ST398 (n = 3; 27% each) and one ST508 were identified in farm B (Supplementary S6). Six additional *S. aureus* sequence types were identified on the farms, including a novel ST, ST8620 (n = 3; 27%) on farm B (Supplementary S6).

ST61 (n = 11; 31%) was the most common sequence type amongst *M. sciuri* in farm A and ST239 (n = 15; 68%) in farm B. Other sequence types identified in *M. sciuri* included eight novel sequence types. All ST61 and ST239 strains harboured *mecA*. Among the other SOSA species, there was a diversity of STs, with a number of novel STs identified in all species; specifically, all the STs amongst the *S. chromogenes* isolates were novel. In total, 29 novel sequence types were identified in this study (Supplementary S6). There is no MLST scheme for *S. hyicus*.

Only SCC*mec*_type_II(2A) and SCC*mec*_type_V(5C2) were identified in *S. aureus* (farm A) and *S. epidermidis* (farm B) respectively. On farm A, 29% of *M. sciuri* isolates had genes/elements which were associated with SCC*mec*_type_III (3A) & SCC*mec*_type_VIII(4A) and were mostly observed in ST61 strains. On both farms all ST239 strains were associated with SCC*mec*_type_III(3A) only.

## Discussion

This study describes the species distribution, antibiotic resistance profiles and strain types in *Staphylococcus* spp. from two commercial pig farms in the Western Cape, South Africa. The staphylococcal isolates from this study are carriage isolates from asymptomatic animals, farm workers and environmental isolates.

There was high diversity of staphylococcal species identified, with *M. sciuri* and *S. hyicus* as the most frequently isolated species from pigs. Certain species of *Staphylococcus,* most commonly *S. hyicus,* are known to cause exudative epidermitis in pigs^31^*.* Although only a small number of staphylococci were isolated from farm workers and the environment, 18 of the 21 *S. epidermidis* were from humans. This is similar to a reports in literature that *S. epidermidis* is the most common SOSA colonizing healthy individuals^32^. This might be an indication that farm workers were colonized outside of the farm setting and that there is a limited transmission of *S. epidermidis* from workers to the pigs.

The rate of colonisation with *S. aureus* in the pigs (11%) is similar to what has been reported elsewhere in South Africa (12%) but higher than reports from Nigeria (5%) and Tanzania (4%)^19,33,34^. The rate of *S. aureus* identified in the farm workers (28%), the environment (17%) and the pigs (11%) confirms what has been described in literature that up to 30% of the human population are asymptomatic carriers of *S. aureus* and are permanently colonised^35^. The proportion of *S. aureus* isolates identified in this study also agrees with a systematic review conducted in Africa which reported that the rate of *S. aureus* in pigs ranges from 0 to 55%^36^. However, data on the environment is lacking.

The most common SOSA identified in pigs were *M. scuiri* (33%) and *S. hyicus* (26%). The prevalence of *M. sciuri* was comparable to a study from Denmark conducted using nasal samples from pigs (40%)^37^. A recent study in Italy also identified *M. sciuri* as a predominant SOSA species in five pig farms^38^. In contrast, prevalence of *S. hyicus* in this study was higher than in pigs in studies from Denmark (16%) and Uganda (6%)^37,39^. This is concerning since *S. hyicus* is known to cause exudative epidermidis in pigs^40^. However, further research is required to understand the high rate of *S. hyicus* colonisation in the pigs in our setting. Although, no *S. hyicus* was identified in humans, the three isolated from the environment could be an indication of dissemination from pigs into the environment.

The prevalence of *S. aureus* including MRSA in pigs in Africa ranges from 0 to 55%^41^. In this study, only one *S. aureus* (7%) isolate was methicillin resistant. The rate observed in this study is surprisingly lower than a previous report from South Africa by Van Lochem et al*.*,^19^. The high rate of resistance to erythromycin (80%), clindamycin (100%) and tetracycline (93%) in *S. aureus* in this study and the high erythromycin (89%), clindamycin (89%) and tetracycline (89%) resistance rates seen in isolates from the environment might be indicative of spread from pigs to the environment. The rate of erythromycin, clindamycin and tetracycline resistance among pigs in this study is higher than that described in a review conducted by Katakweba et al*.*, describing antibiotic-resistant *S. aureus* in pigs in Africa and a nationwide pig study in Switzerland^42^. Interestingly, only one *S. aureus* isolate from the farm workers was non-susceptible to the above antibiotics, which suggests that transfer of resistant organisms from animals to humans is uncommon. It is also not clear what factors may influence the risk of zoonotic transfer.

While *S. aureus* is the most studied staphylococcal species, there is limited data on antibiotic resistance in SOSA globally and in Africa. The rate of methicillin resistance in *M. sciuri* isolates in this study (72%) is lower than what has been reported in Italy (88%)^38^ and in broilers in Turkey (100%)^43^. Clindamycin, erythromycin and tetracycline resistance rates of greater than 90% were observed in *M. sciuri*, consistent with the 99% clindamycin resistance rate reported in *M. sciuri* from Belgium but higher than the rates of erythromycin (52%) and tetracycline (62%) resistance reported by the same Belgian study^44^. The tetracycline resistance rate was also higher than the 29% reported in Brazilian pigs in 2012^45^. All *M. sciuri* isolates from the environment were resistant to clindamycin, erythromycin, and tetracycline. This suggests that *M. sciuri* might be a reservoir of antibiotic resistance genes, and may also indicate movement of isolates from pig to environment (or vice versa).

The high tetracycline resistance rates seen in this study may reflect the use of this antibiotic in pigs in South Africa^46^. Although we could not obtain data on antibiotic usage from the farms in this study, national surveillance data shows that tetracycline is used in large amounts in animals in South Africa; 45% of antimicrobial sales in livestock industry in 2020 were tetracyclines^47^. A recent study by Ocloo et al. showed that 39% of *S. haemolyticus* carriage isolates were resistant to tetracycline, suggesting potential spillage from pigs or livestock^48^.

A study including three countries in Europe showed that 59% of *S. hyicus* from Danish pigs were erythromycin resistant which was the highest in Europe^49^, and is similar to the 51% reported in this study. The rate of clindamycin resistance was 59% among *S. hyicus* isolates, a similar proportion (53%) was reported in diseased pigs in Europe^49^. Clindamycin is used in both clinical and veterinary medicine, making the 100% resistance rate among *M. sciuri* isolates particularly concerning. The primary cause of clindamycin resistance in *M. sciuri* is the presence of the *sal(A)* gene. This gene has been demonstrated to provide intrinsic resistance to both lincosamides and streptogramin A in *M. sciuri*^50^*.* In South Africa, fusidic acid is an antistaphylococcal agent used in the treatment of infections due to staphylococci, it is worrying that 91% of *M. sciuri* isolates were resistant to the antibiotic.

There was only a 60% correlation between phenotypic methicillin resistance and the presence of the *mecA* gene in SOSA. Additionally, the *mecA* gene was observed in only one methicillin susceptible *S. aureus.* No other *mec* genes were detected in the one MRSA isolate*.* Similarly, none of the methicillin resistant *S. aureus* isolated from pigs in Tanzania harboured *mecA*^33^. In *S. epidermidis* methicillin resistance was only seen in one isolate and it harboured *mecA*. In our study 78% of methicillin resistant *M. sciuri* isolates harboured *mecA*. The poor correlation observed in our study between phenotypic methicillin resistance and the presence of *me*cA in SOSA confirms challenges in using cefoxitin for screening for methicillin resistance in SOSA, as described by Yang et al*.*^51^ and Humphries et al*.*^52^.

The tetracycline resistance gene *tetK* is the most common gene identified in tetracycline resistant staphylococcal species isolated from pigs globally^53^, and this study also identified *tetK* as the common gene across all staphylococcal species. The *tetK* gene has been reported to be plasmid-mediated^43^, and this increases the risk of spread into clinical settings. The erythromycin resistance gene *ermC* is the most common gene identified in erythromycin resistant staphylococcal species isolated from pigs globally, and was also identified across all staphylococcal species in this study^54^. The high clindamycin resistance rate seen in this study, in the absence of clindamycin resistance genes, might be due to the expression of *erm* genes^55^.

Some typical human-associated *S. aureus* strains are also found in animals; an example is the ST1 strain^56^. In this study, 53% of *S. aureus* isolates from pigs belonged to ST1, this shows a possible human-animal contamination on the farms or that ST1 has become animal-adapted. This strain has been reported to cause infections in cattle and pigs in Europe^57,58^. The identification of ST398 which is the most common livestock associated strain and a novel ST8620 in pigs, humans and the environment might be an indication of a possible transmission between the selected ecological niches on the farm.

*M. sciuri* and *S. hyicus* are the two most common SOSA identified in this study. The predominance of *M. sciuri* ST61 and ST239 on farm A and farm B respectively, is evidence of clonal spread of different STs within the two farms. ST239 and ST61 are the most common sequence types identified amongst *M. sciuri*, and although these strains are not newly assigned in this study, there are no reports in literature. This demonstrates the lack of published data on the strain typing of SOSA. There is no MLST scheme available for *S. hyicus,* restricting their characterisation.

*M. sciuri* was the only species in this study with diversity of SCC*mec* types; *S. aureus* and *S. epidermidis* harboured one SCC*mec* type each. SCC*mec* type III was the commonest among the methicillin resistant *M. sciuri* isolates. This is consistent with numerous studies where SCC*mec* type III has been the most commonly identified SCC*mec* type in *M. sciuri* from both animals and humans^44,59,60^. It has been hypothesized that SCC*mec* type III originated in *M. sciuri,* and may have been the first SCC*mec* cassette to be assembled, possibly by a recombination event in a human host or a human-created environment, and later was transferred to *S. aureus*^61^*.*

## Conclusion

The study identified a diversity of staphylococcal species in pigs and high rates of tetracycline resistance in pig farms in South Africa, with some evidence of possible sharing of strains and antibiotic resistance genes between pigs and humans. This highlights the need for policy makers to regulate the use antibiotics, and specifically tetracycline in pig farming to reduce the burden of AMR infections in the clinical sector in South Africa.

The study highlights the need for further research on antibiotic resistance and strain typing in SOSA in animals globally and in South Africa, which can help in designing effective antibiotic resistance surveillance and control strategies in animal husbandry.

## Supplementary Information

Supplementary Information 1.

Supplementary Information 2.

Supplementary Information 3.

Supplementary Information 4.

Supplementary Information 5.

Supplementary Information 6.

## Data Availability

Sequence data that support the findings of this study have been deposited in the National Centre for Biotechnology Information with the primary accession code Bioproject; PRJNA1018240.
