# Supplementary material for: Epidemiology and antibiotic resistance of staphylococci on commercial pig farms in Cape Town, South Africa
Source: Sci Rep. 2024 Aug 26;14:19747. doi: 10.1038/s41598-024-70183-2 (PMC11347665; doi:10.1038/s41598-024-70183-2)
Supplement: Supplementary file 1 — Supplementary Information 1. [file 41598_2024_70183_MOESM1_ESM.docx]

**Supplementary_1**

**Questionnaire for farm managers**

**Thank you for participating in this project entitled “Antibiotic Resistant Staphylococci in South Africa-A One Health Approach”. Please kindly note that your participation is voluntary and your information will be anonymous and confidential.**

**Date: __ __/__ __ __ /__ __ __ __**

dd / mm / yyyy

**1). What is the size of the farm?**

o 500 - 2500 m^2^

o 2501 - 5000 m^2^

o >5000 m^2^

o Other. Specify______________________

**2). What is the estimated herd size?**

o 100-500

o 501-1000

o >1000

1. **What proportion are piglets**

o 10-40%

o 40-70%

o 70-90%

o Other. Specify_________________

1. **What proportion are weaners**

o 10-40%

o 40-70%

o 70-90%

o Other. Specify_________________

1. **What proportion are finishers.**

o 10-40%

o 40-70%

o 70-90%

o Other. Specify_________________

o Other. Specify_____________________

**3). How many abattoirs do you supply pigs to?**

o 1-3

o 4-6

o 7-9

o Other. specify_______________________

**4). How are the pig houses/pens located?**

o Adjacent each other

o 1-2m distant from each other

o 3-4m distant from each other

o Other. specify_______________________

**5). How many pigs do you keep per house/pen?**

o 25-50

o 51-100

o 101-200

o Other. Specify_____________________

**6).What is the floor of the house/pen made from?**

o Concrete

o Brick

o Earth

o Other, Specify_______________________

**7). Which age of the pigs do you use bedding for?**

o Piglets

o Weaners

o Finishers

o None

**8). Which type of bedding do you use?**

o Edible

o Non-edible

o Other. Specify___________________

**9). Where do you get food for the pigs?**

o Commercial food.

o Home/market left overs

o Both

o Other. Specify_________________________

**10). How often do you health check the pigs?**

o 1- 3 months

o 4- 6 months

o Yearly

o Only when needed

o Other. Specify__________________________

**11). How do you respond to mildly sick pigs?**

o Isolate them and call the Vet immediately

o Isolate them and wait for a few days before calling the Vet.

o Keep them with the others and call Vet immediately.

o Keep them with the others and wait for a few days before calling the Vet.

o Other. specify________________________

**12). How do you diagnose the pigs?**

o Self

o Veterinarians

o Both

**13). Who treats the pigs?**

o Self

o Veterinarians

o Both

o Other. Specify_______________

**15). On average what proportion of the pigs receive therapeutic antibiotics annually?**

o <25%

o between 25% and 50%

o Between 50% and 75%

o >75%

**16). If you are using an antibiotic to treat a sick animal, do you also administer antibiotics to the pigs that are in the same space as the sick pig?**

o Yes

o No

o I don’t know

o Depending on what the Veterinarians says.

**17). What is used to promote growth in the pigs?**

o Antibiotics

o Probiotics

o I don’t know

o Other specify______________________

**18). How do you impregnate the pigs?**

o Natural

o Veterinarian assisted

o Both

o None

**19). How often do you clean the pig house?**

o Daily

o Weekly

o Only when needed

**20). How often do you change the drinking water of the pigs?**

o Daily

o Weekly

o Only when water is finished

**21). How often do you change feed for the pigs?**

o Daily

o Weekly

o Only when feed is finished

**22). How do you disposed sewage**

o Burial

o Composting/Fertilizer

o Burning

o Other. Specify___________________

1. **Questionnaire for farm workers**

**Thank you for participating in this project entitled “Antibiotic Resistant Staphylococci in South Africa-A One Health Approach”. Please kindly note that your participation is voluntary and your information will be anonymous and confidential.**

**Date: __ __/__ __ __ /__ __ __ __**

dd / mmm / yyyy

1). **How old are you?**

o 18-29 years

o 30-39 years

o 40-49 years

o 50-59 years

o Other. specify age ________________

**2). What is your gender?**

o Male

o Female

o Other

**3). Highest level of Education**

o Left school before matric

o Completed matric

o Certificate

o Diploma/Degree

**4). What sort of dwelling do you live in?**

o formal brick house

o Informal settlement / shack

o Other. specify___________________

1. **How many people do you share the home with?**

o 0-5

o 6-10

o11-15

o Other. specify___________________

1. **Do you have access to piped water in the house?**

o yes

o communal tap

o others

1. **Do you have access to sewage disposal?**

o Toilet in the house

o Communal Toilet

o No formal sewage disposal

**5).How long have you been working on this farm?**

o Less than 6 months

o 6 months to 12 months

o More than 12 months

o Other. specify____________________________

6**). How many hours do you spend on the farm per day?**

o 3-5 hrs

o 6-8 hrs

o 9-12 hrs

o Other. specify__________________________

1. **How many days do you work per week?**

7).**Do you come in contact with the pigs?**

o No

o Yes

**If yes, which pigs do you come in contact with?**

o Piglet’s house

o Weaner’s house

o Finisher’s house

**8). Did you use antibiotics for yourself within the last 3 months?**

o Yes

o No

o Don’t know

**9). Have you been hospitalized (admitted to hospital overnight) within**

**the last 3 months?**

o Yes

o No

o Don’t know

10). **Do you have pets?**

o Yes

o No

If Yes specify________

**11). Which personal protective equipment do you normally wear while working with the pigs?**

o mask

o glove

o gown

o Other. specify_________________

**12). How often do you use hand sanitizers after working with the pigs?**

o Most of the time

o Sometimes

o Hardly evero Not at all.
