# Supplementary material for: Epidemiology and antibiotic resistance of staphylococci on commercial pig farms in Cape Town, South Africa
Source: Sci Rep. 2024 Aug 26;14:19747. doi: 10.1038/s41598-024-70183-2 (PMC11347665; doi:10.1038/s41598-024-70183-2)
Supplement: Supplementary file 2 — Supplementary Information 2. [file 41598_2024_70183_MOESM2_ESM.docx]

**Supplementary 2 | Characteristics of farm A and farm B**

|  | **Farm A** | **Farm B** |
| --- | --- | --- |
| Farm size | 80000m^2^ | 2501-5000m^2^ |
| Herd size | 501-1000 | 501-1000 |
| Age of pigs | finishers | finishers, weaners, piglets |
| Production | Not in production at the time of sampling | Supplies to 1-3 abattoirs |
| Distance between pens | 10m distance | Adjacent/No distance |
| Pigs per pen | 25-50 | 51-100 |
| Bedding | No bedding | Bedding for piglets |
| Isolation of sick pigs | Isolate and wait few days before calling veterinarian | Keep with other healthy animals and wait for few days before calling veterinarian |
| Who treats pigs | Farm manager and veterinarian | Farm manager and veterinarian |
| Proportion of pigs receiving therapeutic antibiotics annually | <25% | >75% |
| Administration of antibiotics to healthy animals in the same space as sick animals | No | Yes |
| Cleaning of pen | Weekly | Daily |
| Changing of feed and water | Pig nibble drinker and ad-lib feeder (automated) | Daily |
| Disposal of sewage | Effluent plant | Compost |
