# Supplementary material for: Epidemiology and antibiotic resistance of staphylococci on commercial pig farms in Cape Town, South Africa
Source: Sci Rep. 2024 Aug 26;14:19747. doi: 10.1038/s41598-024-70183-2 (PMC11347665; doi:10.1038/s41598-024-70183-2)
Supplement: Supplementary file 3 — Supplementary Information 3. [file 41598_2024_70183_MOESM3_ESM.docx]

**Supplementary 3**| *Demographic data of farm workers from farm A compared to farm B.*

|  | **Farm A, n=9 (%)** | **Farm B, n=12 (%)** |
| --- | --- | --- |
| **Gender** |  |  |
| Male | 7 (78) | 8 (67) |
| Female | 2 (22) | 4 (33) |
| **Education** |  |  |
| Pre-Matric | 6 (67) | 8 (67) |
| Matric or Higher | 3 (33) | 4 (33) |
| **Housing** |  |  |
| Formal | 4 (44) | 6 (50) |
| Informal | 5 (56) | 6 (50) |
| **Co-habitation** |  |  |
| Yes | 8 (89) | 12 (100) |
| No | 0 | 0 |
| No Information Provided | 1 (11) |  |
| **Facilities** |  |  |
| Water and Toilet in-house | 5 (56) | 9 (75) |
| Communal Tap and Toilet/No Proper Sewage Disposal | 4 (44) | 2 (17) |
| No Information Provided | 0 | 1 (8) |
| **Contact with Pigs** |  |  |
| Yes | 8 (89) | 11(92) |
| No | 0 | 1 (8) |
| No Information Provided | 1 (11) | 0 |
| **Antibiotic Use in last 3 months** |  |  |
| Yes | 0 | 0 |
| No | 9 (100) | 12 (100) |
| **Pets in household** |  |  |
| Yes | 2 (22) | 2 (17) |
| No | 7 (78) | 10 (83) |
