# Supplementary material for: Epidemiology and antibiotic resistance of staphylococci on commercial pig farms in Cape Town, South Africa
Source: Sci Rep. 2024 Aug 26;14:19747. doi: 10.1038/s41598-024-70183-2 (PMC11347665; doi:10.1038/s41598-024-70183-2)
Supplement: Supplementary file 4 — Supplementary Information 4. [file 41598_2024_70183_MOESM4_ESM.docx]

**Supplementary 4** | Proportion of Antibiotic Resistant *Staphylococcus* spp. among farm workers and the farm environment

| **Environment** | **Species** | **No. of isolates** | **FOX**  **n (%)** | **E**  **n (%)** | **CD**  **n (%)** | **TS**  **n (%)** | **T**  **n (%)** | **LEV**  **n (%)** | **FA**  **n (%)** | **MDR**  **n (%)** |
| --- | --- | --- | --- | --- | --- | --- | --- | --- | --- | --- |
|  | *S. aureus* | 8 | 2 (25) | 7 (88) | 7 (88) | 0 | 8 (100) | 3 (38) | 0 | 6 (75) |
|  | *S. hyicus* | 3 | 3 (100) | 0 | 0 | 0 | 3 (100) | 0 | 0 | 0 |
|  | *S. chromogenes* | 2 | 0 | 2 (100) | 2 (100) | 0 | 2 (100) | 0 | 0 | 2 (100) |
|  | *S. epidermidis* | 1 | 0 | 1 (100) | 1 (100) | 0 | 1 (100) | 0 | 1 (100) | 1 (100) |
|  | *M. sciuri* | 10 | 7 (70) | 5 (50) | 10 (100) | 0 | 10 (100) | 0 | 10 (100) | 10 (100) |
| **Workers** | *S. aureus* | 6 | 2 (33) | 1 (17) | 1 (17) | 0 | 1 (17) | 0 | 0 | 1 (17) |
|  | *S. epidermidis* | 18 | 1 (6) | 4 (22) | 0 | 5 (28) | 12 (67) | 0 | 1 (6) | 1 (6) |
|  | *M. sciuri* | 1 | 0 | 1 (100) | 1 (100) | 0 | 1 (100) | 0 | 1 (100) | 1 (100) |
