# Supplementary material for: Epidemiology and antibiotic resistance of staphylococci on commercial pig farms in Cape Town, South Africa
Source: Sci Rep. 2024 Aug 26;14:19747. doi: 10.1038/s41598-024-70183-2 (PMC11347665; doi:10.1038/s41598-024-70183-2)
Supplement: Supplementary file 5 — Supplementary Information 5. [file 41598_2024_70183_MOESM5_ESM.docx]

**Supplementary 5** | Antibiotic Resistance Genes identified in Staphylococcus species.

| ***S. aureus*** | | ***M. sciuri*** | | ***S. hyicus*** | | ***S. epidermdis*** | | ***S. haemolyticus*** | | ***S. chromogenes*** | |
| --- | --- | --- | --- | --- | --- | --- | --- | --- | --- | --- | --- |
| Antibiotic Resistance Genes | No. of Isolates (%) | Antibiotic Resistance Gene | No. of Isolates (%) | Antibiotic Resistance Gene | No. of Isolates (%) | Antibiotic  Resistance  Gene | No. of Isolates (%) | Antibiotic Resistance Gene | No. of Isolates (%) | Antibiotic Resistance Gene | No. of Isolates (%) |
| *blaZ* | 24 (92) | *ANT(4')-Ib* | 12 (21) | *fusE* | 35 (90) | *blaZ* | 19 (90) | *blaZ* | 3 (20) | *blaZ* | 18 (78) |
| *FosB* | 8 (31) | *ANT(9)-Ia* | 16 (28) | *gyrA* | 2 (5) | *mupA* | 7 (33) | *ANT(6)-Ia* | 1 (7) | *fusE* | 23 (100) |
| *GlpT* | 8 (31) | *dfrG* | 23 (40) | *LmrS* | 2(5) | *AAC(6')-Ie-APH(2'')-Ia bifunctional protein* | 1 (5) | *dfrK* | 2 (13) | *ANT(4')-Ib* | 8 (35) |
| *gyrA* | 12 (46) | *dfrK* | 1 (2) | *parC* | 2 (5) | *ANT(9)-Ia* | 2 (10) | *ErmC* | 2 (13) | *dfrK* | 7 (30) |
| *ileS* | 1 (4) | *ErmA* | 30 (53) | *(blaZ)* | 18 (46) | *chloramphenicol acetyltransferase* | 2 (10) | *lsaE* | 1 (7) | *ErmC* | 6 (26) |
| *norA* | 26 (100) | *ErmB* | 4 (7) | *ANT(4')-Ib* | 13 (33) | *dfrC* | 20 (95) | *mgrA* | 12 (80) | *ErmT* | 12 (52) |
| *parC* | 12 (46) | *ErmC* | 19 (33) | *ANT(9)-Ia* | 3 (8) | *dfrG* | 2 (10) | *norC* | 15 (100) | *norC* | 23 (100) |
| *parE* | 1 (4) | *ErmY* | 23 (40) | *arlR* | 2 (5) | *ErmA* | 2 (10) | *qacG* | 3 (20) | *sdrM* | 23 (100) |
| *ANT(4')-Ib* | 1 (4) | *FosD* | 12 (21) | *arlS* | 2 (5) | *ErmC* | 3 (14) | *qacJ* | 11 (73) | *sepA* | 23 (100) |
| *ANT(9)-Ia* | 12 (46) | *lnuA* | 6 (11) | *dfrC* | 5 (13) | *fusB* | 2 (10) | *sdrM* | 15 (100) | *spd* | 1 (4) |
| *arlR* | 26 (100) | *lnuG* | 1 (2) | *ErmA* | 2 (5) | *mdeA* | 12 (57) | *sepA* | 15 (100) | *spd* | 1 (4) |
| *arlS* | 26 (100) | *mecA* | 31 (54) | *ErmC* | 13 (33) | *mecA* | 1 (5) | *tet(45)* | 1 (7) | *tet(45)* | 3 (13) |
| *dfrG* | 7 (27) | *mecI* | 31 (54) | *ErmT* | 1 (3) | *mgrA* | 21 (100) | *tet(K)* | 10 (67) | *tet(K)* | 9 (39) |
| *ErmA* | 12 (46) | *mecR1* | 31 (54) | *lnuA* | 1 (3) | *mphC* | 2 (10) | *tet(L)* | 4 (27) | *tet(L)* | 11 (48) |
| *ErmC* | 6 (23) | *mphC* | 23 (40) | *lnuG* | 1 (3) | *msrA* | 2 (10) | *tet(T)* | 2(13) | *tet(T)* | 1 (4) |
| *LmrS* | 26 (100) | *msr(I)* | 1 (2) | *mepA* | 2 (5) | *norA* | 21 (100) | *vanT* gene in *vanG* cluster | 15 (100) | *vanT* gene in *vanG* cluster | 23 (100) |
| *mecA* | 1 (4) | *msrA* | 26 (46) | *mepR* | 2 (5) | *norC* | 21 (100) | *vanY* gene in *vanB* cluster | 11 (73) | *vgaALC* | 1 (4) |
| *mecI* | 1 (4) | *qacG* | 30 (53) | *mgrA* | 2 (5) | *qacJ* | 2 (10) | *vanY* gene in *vanF* cluster | 2 (13) |  | |
| *mecR1* | 1 (4) | *qacJ* | 13 (23) | *norA* | 2 (5) | *sdrM* | 21 (100) | *vanY* gene in *vanM* cluster | 2 (13) |  |  |
| *mepA* | 26 (100) | *salA* | 12 (21) | *norC* | 25 (64) | *sepA* | 21 (100) | *vgaALC* | 3 (20) |  |  |
| *mepR* | 26 (100) | *salC* | 45 (79) | *qacG* | 8 (21) | *tet(K)* | 14 (67) |  | |  |  |
| *mgrA* | 26 (100) | *sdrM* | 57(100) | *qacJ* | 20 (51) | *vanY* gene in *vanB* cluster | 3 (14) |  |  |  |  |
| *murA* | 8 (31) | *sepA* | 57(100) | *sdrM* | 36 (92) |  | |  |  |  |  |

**Supplementary 5 cont’d** | Antibiotic Resistance Genes Identified in *Staphylococcus* species.

| ***S. aureus*** | | ***M. sciuri*** | | ***S. hyicus*** | |
| --- | --- | --- | --- | --- | --- |
| Antibiotic Resistance Genes | No. of Isolates (%) | Antibiotic Resistance Genes | No. of Isolates (%) | Antibiotic Resistance Genes | No. of Isolates (%) |
| *norC* | 26 (100) | *spd* | 11 (19) | *sepA* | 36 (92) |
| *qacJ* | 10 (38) | *tet(45)* | 38 (67) | *tet(38)* | 2 (5) |
| *sdrM* | 26 (100) | *tet(K)* | 7 (12) | *tet(45)* | 7 (18) |
| *sepA* | 26 (100) | *tet(L)* | 10 (18) | *tet(K)* | 13 (33) |
| *tet(38)* | 26 (100) | *tet(M)* | 40 (70) | *tet(L)* | 12 (31) |
| *tet(45)* | 2 (8) | *tet(T)* | 17 (30) | *tet(T)* | 15 (38) |
| *tet(K)* | 12 (46) | *vanT* gene in *vanG* cluster | 57 (100) | *vanT* gene in *vanG* cluster | 36 (92) |
| *tet(L)* | 4 (15) | *vanY* gene in *vanF* cluster | 57 (100) | *vgaALC* | 5 (13) |
| *tet(M)* | 3 (12) |  | | | |
