# Supplementary material for: Epidemiology and antibiotic resistance of staphylococci on commercial pig farms in Cape Town, South Africa
Source: Sci Rep. 2024 Aug 26;14:19747. doi: 10.1038/s41598-024-70183-2 (PMC11347665; doi:10.1038/s41598-024-70183-2)
Supplement: Supplementary file 6 — Supplementary Information 6. [file 41598_2024_70183_MOESM6_ESM.docx]

**Supplementary 6 | Sequence types identified in staphylococci isolated from both pig farms.**

| ***S. aureus*** | **n = 26 (%)** |
| --- | --- |
| ST1 | 11 (42) |
| ST9 | 3 (12) |
| ST398 | 3 (12) |
| ST8620* | 3 (12) |
| ST508 | 2 (8) |
| Other: ST36, ST97, ST152, ST5668 | 4 (15) |
| ***M. sciuri*** | **n = 57 (%)** |
| ST239 | 19 (33) |
| ST61 | 12(21) |
| ST259* | 5 (9) |
| ST85 | 3 (5) |
| ST258* | 2 (4) |
| ST86 | 2 (4) |
| ST92 | 2 (4) |
| ST219 | 2 (4) |
| ST65* | 2 (4) |
| Other: ST222, ST225, ST260*, ST261*, ST262*, ST63*, ST264* | 7 (12) |
| ***S. chromogenes*** | **n = 23 (%)** |
| ST158* | 7 (30) |
| ST154* | 3 (13) |
| ST156* | 3 (13) |
| ST159* | 2 (9) |
| Other: ST150*, ST151*, ST152*, ST153*, ST155*, ST157*, ST160*, ST161* | 8 (35) |
| ***S. epidermidis*** | **n = 21 (%)** |
| ST59 | 4 (19) |
| ST57 | 2 (10) |
| ST100 | 2 (10) |
| ST1221* | 2 (10) |
| Other: ST7, ST60, ST89, ST125, ST218, ST549, ST862, ST1222*, ST1223*, ST1224*, ST1225* | 11 (52) |
| ***S. haemolyticus*** | **n = 12 (%)** |
| ST150* | 7 (58) |
| ST149* | 3 (25) |
| Other: ST75, ST151* | 2 (25) |

*Other: ST represented by a single isolate. *Novel sequence types*
